# Luteolin Alleviates the TNF-*α*-Induced Inflammatory Response of Human Microvascular Endothelial Cells via the Akt/MAPK/NF-*κ*B Pathway

**DOI:** 10.1155/mi/6393872

**Published:** 2024-12-11

**Authors:** Qing-Yu Lu, Li Guo, Qi-Yun Zhang, Fu-Mei Yang, Shu-Ting Zhou, Qian-Yun Sun

**Affiliations:** ^1^State Key Laboratory of Functions and Applications of Medicinal Plants, Guizhou Medical University, Guiyang 550014, China; ^2^Institute of Pharmacology and Bioactivity, Natural Products Research Center of Guizhou Province, Guiyang 550014, China

**Keywords:** endothelial cells, inflammation, luteolin, signal pathways, TNF-*α*

## Abstract

Endothelial dysfunction and pathological alterations are pivotal in the pathogenesis of cardiovascular disease. To date, effective interventions for these endothelial changes are lacking. Tumor necrosis factor-alpha (TNF-*α*) is known to significantly contribute to these alterations. It has been reported the potential of luteolin to mitigate TNF-*α*-induced inflammation, yet its specific mechanisms and targets still remain to be elucidated. This study aims to investigate the effects and mechanisms of luteolin on TNF-*α*-induced inflammatory injury in human microvascular endothelial cells, thereby advancing the understanding of luteolin's medicinal properties. Our findings demonstrate that luteolin notably inhibits TNF-*α*-induced phosphorylation of Akt, mitogen activated protein kinase (MAPK), and the nuclear factor-kappaB (NF-*κ*B) p65. It significantly reduces the transcriptional activity of NF-*κ*B p65 and AP-1 and decreases the expression of mRNA and proteins related to adhesion molecules and inflammatory mediators. Additionally, luteolin inhibited the reduction in STAT3 phosphorylation. In conclusion, luteolin effectively suppresses TNF-*α*-induced inflammatory injury in endothelial cells via the Akt/MAPK/NF-*κ*B pathway.

## 1. Introduction

Cardiovascular disease (CVD) has become a significant focus of research due to the impact and devastating effects it has on the world, it is imperative to improve the prevention and treatment of CVD. Many risk factors have been associated with the formation of CVD, but inflammation and cellular dysfunction within the endothelial cells (ECs) play one of the most significant roles in the initiation process [1–4]. Therefore, preventing the inflammation and dysfunction of ECs is an important target for drug development to treat CVD.

ECs act as both target and effector cells in the innate immune system during inflammation [5, 6], which secrete various inflammatory mediators and cytokines, participate in the body's inflammation, and immune response processes. Meanwhile, studies have unequivocally established that the inflammation response of ECs underlies the pathological basis for the onset and progression of CVD [7, 8]. EC activation and dysfunction can be caused by numerous factors, such as high glucose levels, endotoxins, and oxidative stress. Among these factors, tumor necrosis factor-alpha (TNF-*α*) assumes a pivotal role in the process [9]. TNF-*α* is a multifunctional cytokine that is believed to mediate inflammation and immune responses by activating associated signaling pathways, including nuclear factor-kappaB (NF-*κ*B), mitogen activated protein kinase (MAPK), and phosphoinositide 3-kinase (PI3K)/Akt signaling pathway [10, 11]. TNF-*α* pathway activation subsequently leads to increased expression of other cytokines, including TNF-*α* itself [12], which may exacerbate inflammatory processes [13]. Although NF-*κ*B, MAPK, and PI3K/Akt signaling pathways are important for cellular responses, aberrant activation of these signaling pathways can induce chronic inflammation, which participates in the regulation of CVD [14–16]. Since TNF-*α*-induced inflammation is important in the pathogenesis of CVD, search for agents that can attenuate TNF-*α*-induced inflammation in ECs could be an effective strategy to prevent and treat CVD.

Accumulating evidence suggest flavonoids have drawn wide scientific attention because of their diverse health benefits and accumulating epidemiological studies show a positive relationship between flavonoid intake and reduced CVD risk [17–20]. Luteolin, a natural flavonoid, that is usually present in a glycosylated form in celery, green peppers, perillaleaves, and chamomile tea [21] and has been found to possess anti-inflammatory property [22]. Studies collectively suggest that luteolin attenuates TNF-*α*-induced inflammatory responses [23–26], which are primarily associated with the NF-*κ*B or MAPK pathway. Nevertheless, the molecular mechanisms underlying the effects of luteolin in reducing ECs inflammation are not fully understood, and most of these findings have been focused on macrovascular ECs. It is worth highlighting that ECs in different organs exhibit distinct phenotypes and functions, attributed to variations in EC gene expression [27], and considering the critical role of microvascular endothelium cells in chronic inflammation and tissue pathology, we established an inflammation model induced by TNF-*α* in human microvascular endothelial cells (HMECs-1) to further elucidate the mechanism of luteolin.

## 2. Materials and Methods

### 2.1. Regents

Luteolin was procured from Solarbio (Beijing, China), TNF-*α* was sourced from Sinobiological (Beijing, China). Celastrol was acquired from Push Biotechnology (Chengdu, China). The fluorescence-labeled antibody (FITC) against intercellular adhesion molecule 1 (ICAM-1) was obtained from BD Biosciences (San Jose, CA, USA). Enzyme-linked immunosorbent assay (ELISA) kits for ICAM-1, interleukin 6 (IL-6), interleukin 8 (IL-8), and monocyte chemoattractant protein 1 (MCP-1) were purchased from Boster (Wuhan, China).

Phosphorylated and total antibodies against inhibiting kappaB kinase beta (IKK*β*), inhibitor kappa B alpha (I*κ*B*α*), NF-*κ*B p65, protein kinase B (Akt), extracellular signal-regulated kinase (ERK), c-Jun N-terminal kinases (JNKs), p38 MAPKs (p38), c-Fos, c-Jun, Janus kinase 2 (JAK2), signal transducer and activator of transcription-3 (STAT3), PI3K, and *β*-actin were sourced from Cell Signaling Technology (USA), while p-PI3K was obtained from Abcam (UK). The mRNA-specific primers were provided by Sangon Biotechnology (Shanghai, China). The NF-*κ*B plasmid (Cat. No. N1111, Promega, USA), AP-1 plasmid (Cat. No. D2108, Beyotime Biotechnology, Shanghai, China), and a plasmid extraction kit (CW Biotechnology, Jiangsu, China) were used. MCDB131 medium and dimethyl sulfoxide (DMSO) were obtained from Sigma (St. Louis, MO, USA). All other reagents used were of analytical grade.

### 2.2. Cell Culture

HMEC-1 cells were generously provided by Professor Xuebo Hu of Huazhong Agricultural University. These cells were cultivated in MCDB131 supplemented with 10% fetal bovine serum, 1 µg/mL hydrocortisone, and 10 ng/mL epidermal growth factor within a cell culture flask. The culture conditions maintained a temperature of 37°C and a CO_2_ concentration of 5%. Subsequently, cells in the logarithmic growth phase were employed for all subsequent experiments.

### 2.3. ELISA

HMEC-1 cells were seeded at a density of 10^5^ cells/mL in 96-well plates. They were pretreated with varying luteolin concentrations (1, 5, and 10 μM) for a duration of 2 h. Subsequently, TNF-*α* (50 ng/mL) was introduced, and the cells were incubated for 24 h. The levels of ICAM-1, IL-6, IL-8, and MCP-1 in the supernatants were quantified using specific ELISA kits following the manufacturer's instructions. The absorbance at 450 nm was determined using a microplate reader (Bio Tek, USA).

### 2.4. Immunofluorescence Staining

The cells were subjected to three washes with preheated phosphate-buffered saline (PBS). Subsequently, 50 μL of Hoechst 33342 and 50 μL of FITC-conjugated ICAM-1 antibody (diluted 500 times with MCDB131 medium) were added to the cells, followed by incubation at 37°C for 30 min in a dark environment. Afterward, the detection solution was removed, and the cells underwent three additional washes with MCDB131 medium. High-content screening system (Thermo, USA) was employed to capture and analyze the images.

For the assessment of intracellular signaling, HMEC-1 cells were pretreated with luteolin (1, 5, or 10 μM) or celastrol (0.5 μM) for 2 h, followed by incubation with TNF-*α* for 1 h. Subsequently, the cells were fixed with 4% paraformaldehyde for 15 min, permeabilized using 0.1% Triton X-100 for 10 min, and blocked with 5% bovine serum albumin for 30 min. The cells were then incubated with an anti-NF-*κ*B p65 antibody at 4°C overnight. Following this, a FITC-linked secondary antibody was applied for 1 h at room temperature while protecting the samples from light. Nuclei were stained with 4′,6-diamidino-2-phenylindole (DAPI, Beyotime Biotechnology, Shanghai, China) for 15 min. Finally, an antifluorescence quencher (Beyotime Biotechnology, Shanghai, China) was introduced. Washes with PBS were performed three times between each of the aforementioned steps. Image acquisition and analysis were carried out using high-content screening system (Thermo, USA). The results are represented as the ratio of nuclear fluorescence intensity to cytoplasmic fluorescence intensity.

### 2.5. Total RNA Extraction and Quantitative Real-Time Polymerase Chain Reaction

RNA extraction from HMEC-1 cells was performed using an RNA extraction kit (Mei5 Biotechnology, Beijing, China) following the manufacturer's instructions. The purity and concentration of the extracted RNA were determined spectrophotometrically using a NanoDrop instrument (Thermo Fisher Scientific, Carlsbad, USA) at 260/280 nm. Subsequently, 1 µg of RNA was reverse-transcribed into cDNA using a cDNA synthesis kit (Mei5 Biotechnology, Beijing, China) and subjected to amplification via quantitative PCR (qPCR) employing SYBR Green PCR Master Mix (Mei5 Biotechnology, Beijing, China). The primer sequences for qPCR are provided in Table 1. The qPCR protocol involved an initial denaturation at 95°C for 2 min, followed by a two-step PCR process (40 cycles: 95°C for 15 s and 60°C for 15 s), culminating in a dissociation step. Relative changes in mRNA levels were assessed utilizing the 2^−*ΔΔ*Ct^ method, with GAPDH levels serving as the control reference. The primer pairs used are detailed in Table 1.

### 2.6. Dual-Luciferase Reporter Assay

For plasmid preparation, we referred to our previous research [28]. Cells (1 × 10^4^/well) were initially seeded into black 96-well plates and allowed to grow overnight. Subsequently, the cells underwent three washes with warm serum-free MCDB 131 medium. They were then supplemented with 90 μL of MCDB 131 medium containing 10% FBS and subjected to transfection using the Liposome Transfection Kit (Beyotime Biotechnology, Shanghai, China) following the provided instructions. The transfection mixture comprised NF-*κ*B or AP-1 expression plasmid, an internal reference plasmid, and liposome reagent, which were added to each well for a 16 h incubation.

Afterward, the supernatant was removed, and 90 μL of MCDB 131 medium, along with 10 μL of varying concentrations of luteolin or warm PBS, was introduced for a 2 h incubation period. Subsequently, 10 μL of cell supernatant was extracted, and 10 μL of TNF-*α* was added for a 3 h incubation. The mixed incubates of PBS and medium served as the negative control. The fluorescence intensity was measured using the Dual-Luciferase Reporter Assay System (Cat. No. E1910, Promega, USA), and the relative nuclear transcription activity (Ra) was calculated utilizing the following formula:

  $$\begin{array}{c} \text{Ra}=\left(R_{1}\text{ treat}/R_{2}\text{ treat}\right)/\left(R_{1}\text{ control}/R_{2}\text{ control}\right)\times 100, \end{array}$$

where *R*_1_ represents the value of firefly luciferase and *R*_2_ represents the value of Renilla luciferase.

### 2.7. Western Blot Analysis

For western blot analysis, total protein was extracted from HMEC-1 cells using radioimmunoprecipitation assay buffer (Beyotime Biotechnology, Shanghai) containing 1% phenylmethylsulfonyl fluoride. The protein concentration was determined using a BCA protein assay kit (Beyotime Biotechnology, Shanghai). The proteins were separated on 10% SDS gels and subjected to polyacrylamide gel electrophoresis. Subsequently, they were transferred onto PVDF membranes (Merck Millipore, Germany).

The PVDF membranes were blocked with a suitable blocking buffer (QuickBlock blocking buffer for western blot analysis, Beyotime Institute of Biotechnology) for 30 min at room temperature. Following the blocking step, the membranes were incubated overnight at 4°C with specific primary antibodies, including p-IKK*β*, IKK*β*, p-I*κ*B*α*, I*κ*B*α*, p-NF-кB p65, NF-кB p65, p-ERK, ERK, p-JNK, JNK, p-p38, p38, p-c-Jun, c-Jun, p-c-Fos, c-Fos, p-Akt, Akt, p-PI3K, PI3K, p-JAK2, JAK2, p-STAT3, STAT3, and *β*-actin. All primary antibodies, except for p-STAT3, were diluted 1000-fold, and p-STAT3 was diluted 2000-fold. After primary antibody incubation, the membranes were probed with a goat antirabbit horseradish peroxidase-conjugated IgG secondary antibody (1:1000, Cell Signaling Technology, USA) at room temperature for 1 h. Finally, protein bands were visualized using enhanced chemiluminescence reagents (Beyotime Biotechnology). Western blot analysis results were quantified by measuring the relative intensity compared to the control using VisionCapt v16.15 software (Vilber Lourmat, France).

### 2.8. Statistical Analysis

The measured data were presented as the mean ± standard deviation based on data collected from at least three independent experiments. GraphPad Prism 8.0 software (GraphPad Software, Inc.) was utilized for graph plotting. Data analysis was carried out using SPSS 18.0. One-way analysis of variance was employed to assess significant differences among the samples, followed by Tukey's test for post hoc comparisons of means. Statistical significance was established when *p* < 0.05.

## 3. Results

### 3.1. Luteolin Inhibited Proinflammatory Cytokine Expression

**Figure 1:**
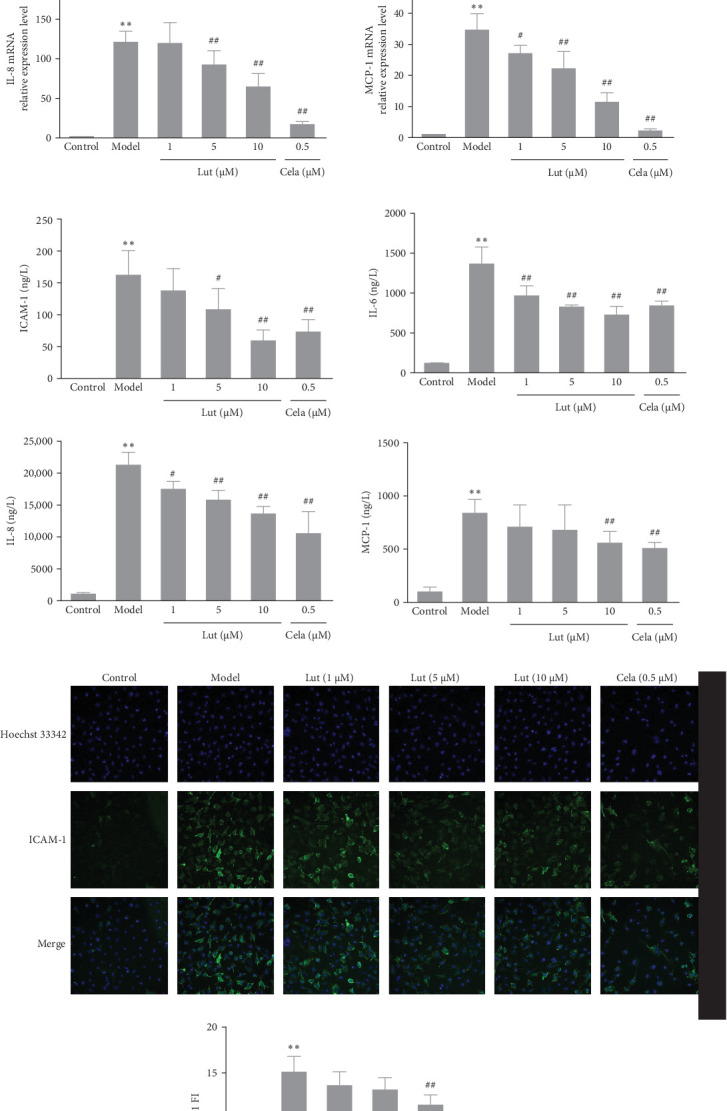
Luteolin inhibited proinflammatory cytokine expression in TNF-*α*-induced HMEC-1 cells. Cells were treated with different concentrations (1, 5, and 10 μmol/L) of luteolin or celastrol (0.5 μmol/L) for 2 h and then incubated with TNF-*α* (50 μg/L). (A) ICAM-1, IL-6, IL-8, and MCP-1 mRNA levels were analyzed with RT‒qPCR after HMEC-1 cells were incubated with TNF-*α* for 3 h. (B) ELISA analysis of ICAM-1, IL-6, IL-8, and MCP-1 protein concentrations in the culture supernatant of HMEC-1 cells incubated with TNF-*α* for 24 h. (C) The expression of ICAM-1 on the cell surface was determined by an immunofluorescence assay after HMEC-1 cells were incubated with TNF-*α* for 24 h. ICAM-1 was stained with an anti-ICAM-1 antibody (green), the nucleus protein was stained with DAPI (blue), and the merged image represents the combined image of ICAM-1 fluorescence and nucleus staining. Images were generated on an HCS (magnification × 200). (D) The semiquantitative results of (C), FI: fluorescence intensity, ICAM-1 FI = the FI of the treatment group/the FI of the control group; the treatment groups included the model, luteolin, and celastrol groups. Values are expressed as the mean ± SD (*n* = 3). *⁣*^*∗∗*^*p* < 0.01, compared with the control group; ^#^*p* < 0.05, ^##^*p* < 0.01, compared with the model group. ELISA, enzyme-linked immunosorbent assay; HMEC-1, human microvascular endothelial cell 1; ICAM-1, intercellular adhesion molecule 1; MCP-1, monocyte chemoattractant protein 1; TNF-*α*, tumor necrosis factor-alpha.

TNF-*α* plays a pivotal role in the pathogenesis of inflammatory diseases by upregulating the expression of various proinflammatory cytokines. Consequently, we utilized TNF-*α* to induce inflammation in ECs and investigated the effect of luteolin on representative proinflammatory cytokines. Our results revealed that TNF-*α* stimulation led to a significant increase in the mRNA levels of ICAM-1, IL-6, IL-8, and MCP-1. However, luteolin treatment resulted in the downregulation of ICAM-1, IL-6, IL-8, and MCP-1 mRNA expression compared to the model group (Figure 1A).

Furthermore, the protein levels of ICAM-1, IL-6, IL-8, and MCP-1 in the culture supernatants were also reduced upon incubation with luteolin in TNF-*α*-induced HMEC-1 cells (Figure 1B). Additionally, an immunofluorescence assay was employed to assess the expression of ICAM-1 on the surface of HMEC-1 cells, as depicted in Figure 1C,D. In comparison to the control group, the model group exhibited a significant increase in ICAM-1 expression (indicated by positive-stained green fluorescence). However, treatment with luteolin at a concentration of 10 μM effectively reduced the intensity of positively stained fluorescence relative to the model group. In light of these findings, luteolin plays a crucial role in attenuating the expression of ICAM-1, IL-6, IL-8, and MCP-1.

### 3.2. Luteolin Inhibited NF-*κ*B Signaling Phosphorylation

**Figure 2:**
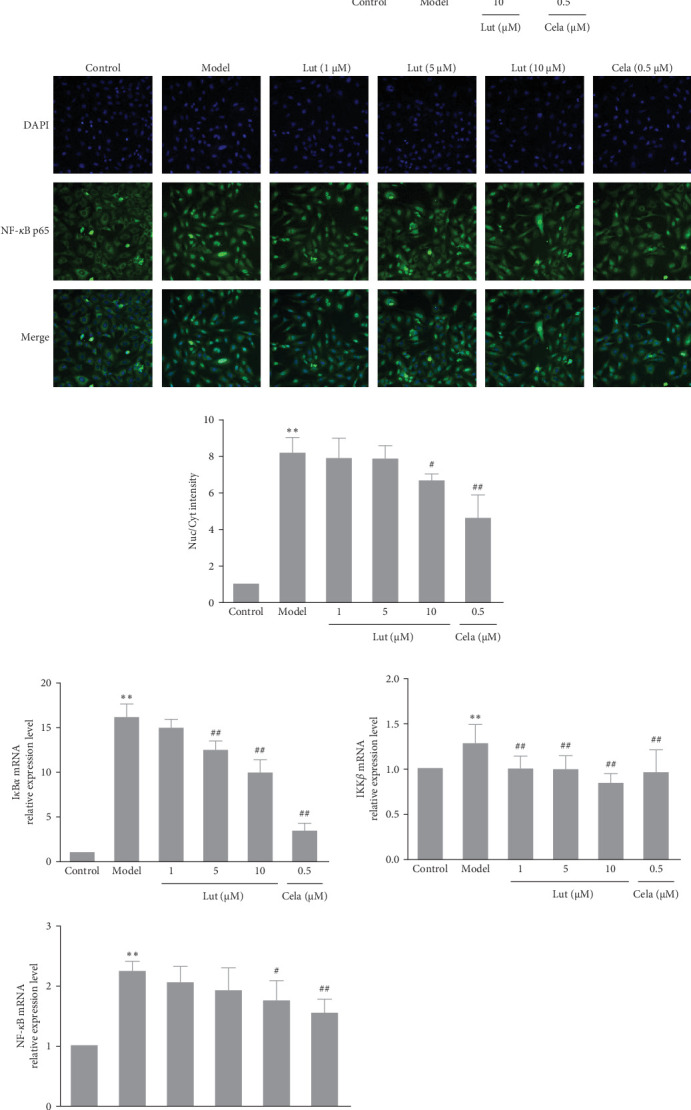
Luteolin inhibited NF-*κ*B signaling phosphorylation in TNF-*α*-stimulated HMEC-1 cells. Cells were treated with different concentrations (1, 5, and 10 μmol/L) of luteolin or celastrol (0.5 μmol/L) for 2 h and then incubated with TNF-*α* (50 μg/L). (A) NF-*κ*B p65 transcriptional activity was detected by a dual-luciferase reporter assay after HMEC-1 cells were incubated with TNF-*α* for 3 h. (B) Protein levels of p-p65 and p65 after HMEC-1 cells were incubated with TNF-*α* for 10 min, *β*-actin was used as an internal control. (C) NF-*κ*B p65 nuclear translocation was analyzed by immunofluorescence after HMEC-1 cells were incubated with TNF-*α* for 1 h. NF-*κ*B p65 was stained with an anti-p65 antibody, the nucleus protein was stained with DAPI (blue), and the merged image shows p65 fluorescence and nucleus staining. Images were generated on a HCS (magnification × 200). (D) The semiquantitative result of (C), nuc/cyt = the FI of nucleus/the FI of cytoplasm; the nuc/cyt ratio was normalized to the control group for each group. (E) mRNA expression of I*κ*B*α*, IKK*β*, and NF-*κ*B was measured by RT-qPCR after HMEC-1 cells were incubated with TNF-*α* for 3 h. GAPDH was used as an internal control. (F) Protein levels of p-I*κ*B*α*, I*κ*B*α*, and IKK*β* were analyzed by western blot after HMEC-1 cells were incubated with TNF-*α* for 10 min, and *β*-actin was used as an internal control. Values are expressed as the mean ± SD (*n* = 3). *⁣*^*∗∗*^*p* < 0.01, compared with the control group; ^#^*p* < 0.05, ^##^*p* < 0.01, compared with the model group. HMEC-1, human microvascular endothelial cell 1; TNF-*α*, tumor necrosis factor-alpha.

NF-*κ*B plays a central role in regulating proinflammatory mediators in inflammatory diseases. Therefore, we investigated the effect of luteolin on TNF-*α*-induced activation of NF-*κ*B signaling. Exposure of HMEC-1 cells to TNF-*α* for 3 h significantly elevated NF-*κ*B transcriptional activity. However, luteolin at concentrations of 5 and 10 μM effectively inhibited the heightened NF-*κ*B activity induced by TNF-*α* (Figure 2A).

Moreover, TNF-*α* stimulation prominently increased NF-*κ*B p65 phosphorylation in HMEC-1 cells, which was substantially reversed by pretreatment with 10 μM luteolin (Figure 2B). Immunofluorescence analysis was employed to investigate the effect of luteolin on NF-*κ*B p65 nuclear translocation. TNF-*α* enhanced NF-*κ*B p65 nuclear translocation, and this effect was partially attenuated by luteolin treatment (Figure 2C).

It is well established that inhibitors of kappa B kinase (IKK), especially IKK*β*, are essential upstream regulators of NF-*κ*B activation. IKK*β* phosphorylates the inhibitory I*κ*B*α* protein, leading to its ubiquitination and subsequent degradation, which results in the dissociation of I*κ*B*α* from NF-*κ*B. To gain further insight into how luteolin inhibits the nuclear translocation of NF-*κ*B, we employed RT‒qPCR and western blotting to examine the effect of luteolin on the expression of I*κ*B-*α*, IKK*β*, and NF-*κ*B mRNA, as well as the levels of total and phosphorylated I*κ*B-*α* and IKK*β*.

The results demonstrated that TNF-*α* induction significantly promoted I*κ*B*α* degradation and phosphorylation of I*κ*B-*α* and IKK*β* and increased the expression levels of I*κ*B-*α* and IKK*β* mRNA in HMEC-1 cells. However, luteolin treatment resulted in a reduction in the levels of I*κ*B-*α*, IKK*β*, and NF-*κ*B mRNA (Figure 2E). Furthermore, we observed no significant differences among any of the treatment groups regarding I*κ*B*α* degradation or phosphorylation of I*κ*B-*α* and IKK*β* in TNF-*α*-induced HMEC-1 cells (Figure 2F).

### 3.3. Luteolin Inhibited MAPK Pathway Phosphorylation

The activation of MAPK signaling plays a central role in the regulation of inflammatory responses by controlling the expression of chemokines and leukocyte adhesion molecules. Consequently, we examined the effect of luteolin on TNF-*α*-induced activation of MAPK signaling.

**Figure 3:**
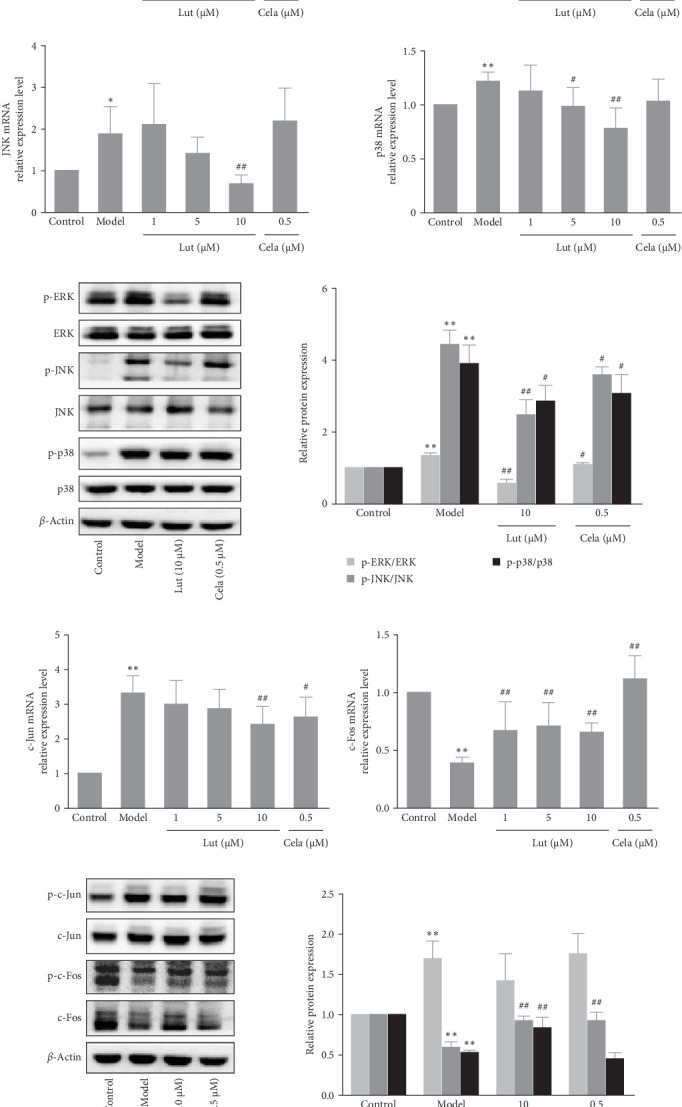
Luteolin inhibited MAPK pathway phosphorylation in TNF-*α*-stimulated HMEC-1 cells. Cells were treated with different concentrations (1, 5, and 10 μmol/L) of luteolin or celastrol (0.5 μmol/L) for 2 h and then incubated with TNF-*α* (50 μg/L). (A) AP-1 transcriptional activity and mRNA levels of ERK, JNK, and p38 were detected by dual-luciferase reporter assay and RT-qPCR after HMEC-1 cells were incubated with TNF-*α* for 3 h. (B) Protein levels of p-ERK, ERK, p-JNK, JNK, p-p38, and p38 were analyzed after HMEC-1 cells were incubated with TNF-*α* for 10 min, and *β*-actin was used as an internal control. (C) mRNA levels of c-Jun and c-Fos were detected by RT‒qPCR after HMEC-1 cells were incubated with TNF-*α* for 3 h. GAPDH was used as an internal control. (D) The protein levels of p-c-Jun, c-Jun, p-c-Fos, and c-Fos were analyzed by western blot after HMEC-1 cells were incubated with TNF-*α* for 12 h, and *β*-actin was used as an internal control. Values are expressed as the mean±SD (*n* = 3). *⁣*^*∗*^*p* < 0.05, *⁣*^*∗∗*^*p* < 0.01, compared with the control group; ^#^*p* < 0.05, ^##^*p* < 0.01, compared with the model group. HMEC-1, human microvascular endothelial cell 1; TNF-α, tumor necrosis factor-alpha.

When HMEC−1 cells were exposed to TNF-*α* for 3 h, there was a potent increase in AP-1 transcriptional activity (Figure 3A). However, luteolin treatment effectively inhibited this elevated AP-1 activity. Subsequently, we assessed the mRNA expression levels of AP-1-related genes, revealing that TNF-*α* induced the upregulation of c-Jun and the downregulation of c-Fos, which was partially reversed by luteolin (Figure 3C). Additionally, we examined the levels of phosphorylated and total c-Jun and c-Fos proteins. The results demonstrated that TNF-*α* induction led to increased phosphorylation of c-Jun and decreased phosphorylation and total expression of c-Fos protein. Luteolin treatment effectively counteracted the effect on c-Fos (Figure 3D).

To investigate the mechanism of luteolin action, we assessed the mRNA and phosphorylation levels of ERK, JNK, and p38. Exposure to TNF-*α* resulted in an upregulation of ERK, JNK, and p38 mRNA expression, and luteolin treatment significantly downregulated the mRNA expression compared to the model group (Figure 3A). A similar pattern was observed for the phosphorylation of ERK, JNK, and p38 proteins (Figure 3B). Luteolin treatment resulted in a significant decrease in the phosphorylation levels of ERK, JNK, and p38 proteins compared to the model group. These findings indicate that luteolin mitigates inflammation induced by TNF-*α*, in part due to its inhibitory effect on MAPK phosphorylation.

### 3.4. The Effect of Luteolin on the PI3K/Akt Pathway

**Figure 4:**
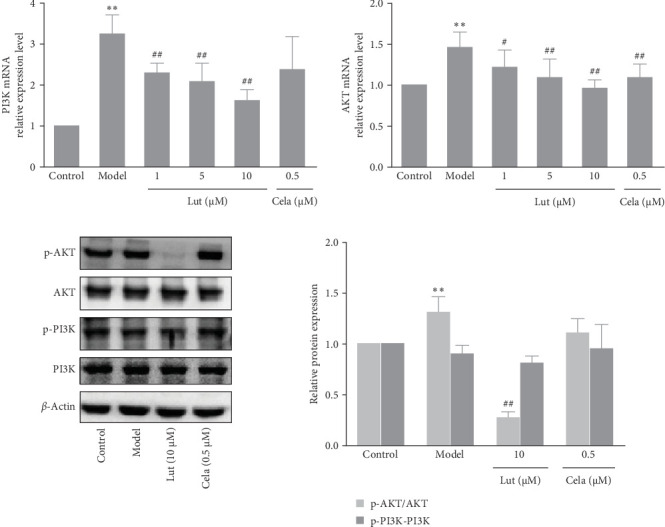
The effect of luteolin on the PI3K/Akt pathway. Cells were treated with different concentrations (1, 5, and 10 μmol/L) of luteolin or celastrol (0.5 μmol/L) for 2 h and then incubated with TNF-*α* (50 μg/L). (A) mRNA levels of PI3K and Akt were detected by RT-qPCR after HMEC-1 cells were incubated with TNF-*α* for 3 h. GAPDH was used as an internal control. (B) Protein levels of p-PI3K, PI3K, p-Akt, and Akt were analyzed by western blot after HMEC-1 cells were incubated with TNF-*α* for 10 min, and *β*-actin was used as an internal control. Values are expressed as the mean ± SD (*n* = 3). *⁣*^*∗∗*^*p* < 0.01, compared with the control group; ^#^*p* < 0.05, ^##^*p* < 0.01, compared with the model group. HMEC-1, human microvascular endothelial cell 1; PI3K, phosphoinositide 3-kinase; TNF-α, tumor necrosis factor-alpha.

The mRNA expression levels of PI3K and Akt were assessed using quantitative reverse transcription–polymerase chain reaction. The results revealed that TNF-*α* induced upregulation of PI3K and Akt mRNA expression (Figure 4A). However, luteolin treatment partially reversed these effects in a concentration-dependent manner. Furthermore, we conducted western blot analysis to examine the phosphorylation levels of PI3K and Akt proteins, as depicted in Figure 4B. Notably, luteolin at a concentration of 10 µM significantly attenuated the TNF-*α*-induced phosphorylation of Akt.

### 3.5. The Effect of Luteolin on the JAK2/STAT3 Pathway

**Figure 5:**
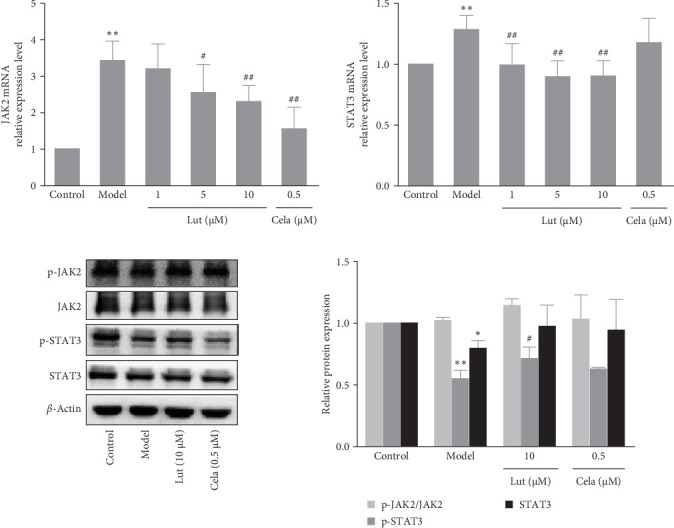
The effect of luteolin on the JAK2/STAT3 pathway after HMEC-1 cells were exposed to TNF-*α*. Cells were treated with different concentrations (1, 5, and 10 μmol/L) of luteolin or celastrol (0.5 μmol/L) for 2 h and then incubated with TNF-*α* (50 μg/L). (A) mRNA levels of JAK2 and STAT3 were detected by RT‒qPCR after HMEC-1 cells were incubated with TNF-*α* for 3 h. GAPDH was used as an internal control. (B) Protein levels of p-JAK2, JAK2, p-STAT3, and STAT3 were analyzed by western blot after HMEC-1 cells were incubated with TNF-*α* for 12 h, and *β*-actin was used as an internal control. Values are expressed as the mean ± SD (*n* = 3). *⁣*^*∗*^*p* < 0.05, *⁣*^*∗∗*^*p* < 0.01, compared with the control group; ^#^*p* < 0.05, ^##^*p* < 0.01, compared with the model group. HMEC-1, human microvascular endothelial cell 1; TNF-*α*, tumor necrosis factor-alpha.

To assess the effects of luteolin on the JAK2/STAT3 signaling pathway, we examined the mRNA expression levels of JAK2 and STAT3 using quantitative real-time PCR. The results demonstrated that TNF-*α* induced the upregulation of JAK2 and STAT3 mRNA expression (Figure 5A). However, treatment of the cells with luteolin inhibited these effects, and the extent of inhibition was positively correlated with the concentration of luteolin. Furthermore, we conducted western blot analysis to assess the expression levels of proteins in the JAK2/STAT3 pathway. The results showed that TNF-*α* induced a decrease in the phosphorylated and total levels of STAT3 protein expression. However, luteolin treatment effectively reversed these effects (Figure 5B).

## 4. Discussion

In this study, we established an inflammation model in human microvascular cells stimulated by TNF-*α*. The results demonstrated a significant upregulation in the mRNA and protein levels of adhesion molecules and inflammatory mediators. Moreover, we observed the phosphorylation of the Akt, NF-*κ*B, and MAPK signaling pathways. Upon treatment with luteolin, potent downregulation of the expression of these adhesion molecules and inflammatory mediators was observed. Luteolin treatment also inhibited the phosphorylation of Akt, MAPK, and NF-*κ*B, thereby effectively suppressing the inflammatory reactions induced by TNF-*α*.

Our findings in this study indicated a substantial reduction in the mRNA and protein expression of ICAM-1, IL-6, IL-8, and MCP-1 upon luteolin treatment. Adhesion molecules and chemokines play a crucial role in initiating and advancing inflammation [29]. Specifically, ICAM-1 and MCP-1 enhance EC-monocyte interactions and subsequently trigger inflammatory responses in ECs [30]. Similarly, IL-6 and IL-8 drive the immune system and influence the course of inflammatory responses [31]. This suggests that luteolin significantly downregulated the transcriptional activity of ICAM-1, IL-6, IL-8, and MCP-1, subsequently affecting their protein expression levels and leading to the effective inhibition of TNF-*α*-induced inflammatory responses. Notably, the changes in the transcriptional levels of these adhesion molecules and inflammatory mediators were consistent with their translational levels, emphasizing the importance of regulating, and activating upstream pathways in the intervention and inhibition of inflammation. Previous evidence has indicated that the NF-*κ*B, MAPK, Akt, and JAK2/STAT3 pathways are closely associated with inflammation regulation [32, 33]. Consequently, these signaling pathways were investigated in our study.

To identify pathways targeted by luteolin, we first investigated NF-*κ*B signaling pathway, a common activator pathway and major therapeutic target in inflammatory diseases. Studies have reported that luteolin ameliorates TNF-*α*-induced inflammation in HUVECS or EA.hy926 cells by blocking the NF-*κ*B pathway, inhibiting I*κ*B degradation, and preventing NF-*κ*B DNA binding, thus inhibiting NF-*κ*B-mediated gene expression and proinflammatory cytokine production [15, 16]. In the present study, luteolin notably suppressed TNF-*α*-induced phosphorylation and nuclear translocation of NF-*κ*B p65, leading to reduced NF-*κ*B p65 transcriptional activity in HMECS, this effect of luteolin on TNF-*α* induced microvascular ECs inflammatory response is similar to the previous studies, namely luteolin inhibited the NF-*κ*B pathway. However, it is worth noting that luteolin did not affect TNF-*α*-induced I*κ*B*α* degradation or IKK*β* phosphorylation in our study. These results suggest that the differences observed may be attributed to cell phenotype differences between the macrovascular ECs and microvascular ECs. NF-*κ*B activation plays a central role in the initiation and development of inflammation, regulating the expression of proinflammatory factors, transcription factors, and adhesion molecules such as ICAM-1, IL-6, and MCP-1 [34, 35]. These results strongly suggest that luteolin exerts its anti-inflammatory properties in part by inhibiting phosphorylation and transcriptional stage of NF-*κ*B p65.

The inflammatory response is a complex network that involves multiple signaling cascades, NF-*κ*B signaling pathway is tightly linked with MAPK, PI3K/Akt, and JAK2/STAT3 signaling pathways. Therefore, we investigated the effects of luteolin on other signaling pathways. We observed luteolin treatment resulted in the inhibition of TNF-*α*-mediated phosphorylation of p38 MAPK, ERK1/2, and JNK, the result is consistent with the previous study [24]. In addition, we further found that luteolin downregulated the AP-1 transcriptional activity. AP-1 as a key factor in the development of CVD. It is a heterodimeric protein composed of proteins belonging to the ATF and JDP, c-Jun, c-Fos families, which could modulate the expression of gene in response to stimuli, such as TNF-*α*. One of the mechanisms is the MAPK signaling pathway, after being phosphorylated, the c-Fos and c-jun proteins will be activated, following the activation of AP-1 [36, 37]. In our study, we observed a decrease in c-Jun mRNA expression, but without affecting c-Jun phosphorylation following luteolin treatment. Interestingly, we found that the phosphorylation, mRNA, and protein expression of c-Fos were markedly reduced after HMEC-1 exposure to TNF-*α* for 12 h, and these downregulated expressions of c-Fos were improved following luteolin intervention. These results suggest that luteolin reduces inflammation by inhibiting the phosphorylation of MAPK and subsequently ameliorating the abnormal expression of c-Fos.

PI3K/Akt signaling pathway is reportedly involved in various cellular processes, including cell cycle progression, proliferation, and survival in response to extracellular stimuli, and it also plays a crucial role in monocyte recruitment, which is linked to the development of several vascular diseases [38]. In our study, we found that TNF-*α* induced an increase in p-Akt phosphorylation and mRNA level, while luteolin treatmemt decreased the phosphorylation and mRNA level of AKT. This result suggests that the protective role of luteolin is linked to the regulation of the AKT pathway. The Akt signaling pathway is reportedly involved in TNF-*α*-induced upregulation of ICAM-1 expression, and expression of constitutively active Akt was sufficient to induce NF-*κ*B activation and ICAM-1 expression in ECs [39]. Study also showed that AKT mediates the activation of NF-*κ*B through PI3K/Akt and TRAF2/NIK signal induced by TNF-*α* [40]. Moreover, the two-stage kinases upstream of the MAPK family members, the MAPK family members and their downstream kinases participate in the activation of I*κ*B*α*, leading to NF-*κ*B activation [41]. TNF-*α* can activate p38, ERKl/2, and NF-*κ*B through TNFRl, inducing the synthesis of IL-6 and IL-8 [42]. These results and reports suggest that luteolin inhibits the phosphorylation of NF-*κ*B may be through the AKT and MAPK signal pathway. Interestingly, following TNF-*α* induction, we did not detect the phosphorylation of JAK2, but we observed a downregulation in STAT3 phosphorylation compared to the control group. However, luteolin treatment partly restored the level of STAT3 phosphorylation. It is well-known that the JAK-STAT pathway cannot be activated directly by TNF-*α*, but STAT3 can be activated by the MAPK signaling pathway. It is worth noting that while ERK2 phosphorylates the serine site of STAT3, thus inhibit the tyrosine site of STAT3 phosphorylated by JAK2 or Src kinases [43], therefore, ERK2 can inhibit the activity of STAT3 in certain signal transduction processes, which may be the reason for the downregulation of STAT3 phosphorylation.

Based on these findings, we infer that luteolin's mechanism of inhibiting TNF-*α*-induced inflammation in HMEC-1 cells is related to Akt/MAPK/NF-*κ*B phosphorylation or its upstream target. However, the precise molecular target still remains unknown and needs further investigation.

## 5. Conclusion

Luteolin effectively inhibits the TNF-*α*-induced inflammatory response in microvascular cells, and its mechanism is highly associated with the inhibition of the phosphorylation of NF-*κ*B, MAPK, and Akt signaling pathways. These results suggest that luteolin is a potential candidate for intervention inflammation.

## Figures and Tables

**Table 1:** Primer sequences for RT-qPCR.

Gene	Forward primer (5–3′)	Reverse primer (5–3′)
GAPDH	GTCTCCTCTGACTTCAACAGCG	ACCACCCTGTTGCTGTAGCCAA
IKK*β*	ACAGCGAGCAAACCGAGTTTGG	CCTCTGTAAGTCCACAATGTCGG
I*κ*B*α*	TCCACTCCATCCTGAAGGCTAC	CAAGGACACCAAAAGCTCCACG
NF-*κ*B p65	TGAACCGAAACTCTGGCAGCTG	CATCAGCTTGCGAAAAGGAGCC
ERK 2	ACACCAACCTCTCGTACATCGG	TGGCAGTAGGTCTGGTGCTCAA
JNK1	GACGCCTTATGTAGTGACTCGC	TCCTGGAAAGAGGATTTTGTGGC
P38	GAGCGTTACCAGAACCTGTCTC	AGTAACCGCAGTTCTCTGTAGGT
c-Jun	GCCTCTCTTACTACCACTCACC	AGATGGCAGTGACCGTGGGAAT
c-Fos	CCTTGAAAGCTCAGAACTCGGAG	TGCTGCGTTAGCATGAGTTGGC
PI3K	CTGTGCCTTCTGCCTTACGGTG	GCAATCGTCGTGGCGTCCTTC
Akt	GGCAGGAGGAGGAGACGATGG	TTCATGGTCACACGGTGCTTGG
JAK2	CCAGATGGAAACTGTTCGCTCAG	GAGGTTGGTACATCAGAAACACC
STAT3	CTTTGAGACCGAGGTGTATCACC	GGTCAGCATGTTGTACCACAGG
ICAM-1	AGCGGCTGACGTGTGCAGTAAT	TCTGAGACCTCTGGCTTCGTCA
IL-6	AGACAGCCACTCACCTCTTCAG	TTCTGCCAGTGCCTCTTTGCTG
IL-8	GAGAGTGATTGAGAGTGGACCAC	CACAACCCTCTGCACCCAGTTT
MCP-1	CCCCAGTCACCTGCTGTTAT	TGGAATCCTGAACCCACTTC

Abbreviations: IκBα, inhibitor kappa B alpha; ICAM-1, intercellular adhesion molecule 1; IKK*β*, inhibiting kappaB kinase beta; JAK2, Janus kinase 2; MCP-1, monocyte chemoattractant protein 1; PI3K, phosphoinositide 3-kinase.

## Data Availability

The data used to support the findings of this study are included within the article.
